# Characterization of phenylpropanoid pathway genes within European maize (*Zea mays *L.) inbreds

**DOI:** 10.1186/1471-2229-8-2

**Published:** 2008-01-03

**Authors:** Jeppe Reitan Andersen, Imad Zein, Gerhard Wenzel, Birte Darnhofer, Joachim Eder, Milena Ouzunova, Thomas Lübberstedt

**Affiliations:** 1 Department of Genetics and Biotechnology, University of Aarhus, Research Center Flakkebjerg, 4200 Slagelse, Denmark; 2 Department of Agronomy and Plant Breeding, Technical University of Munich, Am Hochanger 2, 85354 Freising-Weihenstephan; Germany; 3 Bavarian State Research Center for Agriculture, Vöttinger Str. 38, 85354 Freising-Weihenstephan, Germany; 4 KWS Saat AG, Grimsehlstr. 31, 37555 Einbeck, Germany

## Abstract

**Background:**

Forage quality of maize is influenced by both the content and structure of lignins in the cell wall. Biosynthesis of monolignols, constituting the complex structure of lignins, is catalyzed by enzymes in the phenylpropanoid pathway.

**Results:**

In the present study we have amplified partial genomic fragments of six putative phenylpropanoid pathway genes in a panel of elite European inbred lines of maize (*Zea mays *L.) contrasting in forage quality traits. Six loci, encoding C4H, 4CL1, 4CL2, C3H, F5H, and CAD, displayed different levels of nucleotide diversity and linkage disequilibrium (LD) possibly reflecting different levels of selection. Associations with forage quality traits were identified for several individual polymorphisms within the *4CL1*, *C3H*, and *F5H *genomic fragments when controlling for both overall population structure and relative kinship. A 1-bp indel in *4CL1 *was associated with *in vitro *digestibility of organic matter (IVDOM), a non-synonymous SNP in *C3H *was associated with IVDOM, and an intron SNP in *F5H *was associated with neutral detergent fiber. However, the *C3H *and *F5H *associations did not remain significant when controlling for multiple testing.

**Conclusion:**

While the number of lines included in this study limit the power of the association analysis, our results imply that genetic variation for forage quality traits can be mined in phenylpropanoid pathway genes of elite breeding lines of maize.

## Background

Maize (*Zea mays *L.) is widely used as a silage crop in European dairy agriculture. While breeding efforts in recent decades have substantially increased whole plant yield, there has been a decrease in cell wall digestibility, and consequently feeding value, of elite silage maize hybrids [1,2]. Digestibility of cell walls of forage crops is influenced by several factors, including the content and composition of lignins [3]. Lignins are complex phenolic polymers derived mainly from three hydroxycinnamyl alcohol monomers (monolignols): *p*-coumaryl-, coniferyl-, and sinapyl alcohol. *p*-hydroxyphenyl- (H), guaiacyl- (G), and syringyl units (S), respectively, are derived from these alcohols and polymerize by oxidation to form lignins. In monocots, lignins are predominantly comprised of G and S units [4].

**Figure 1:**
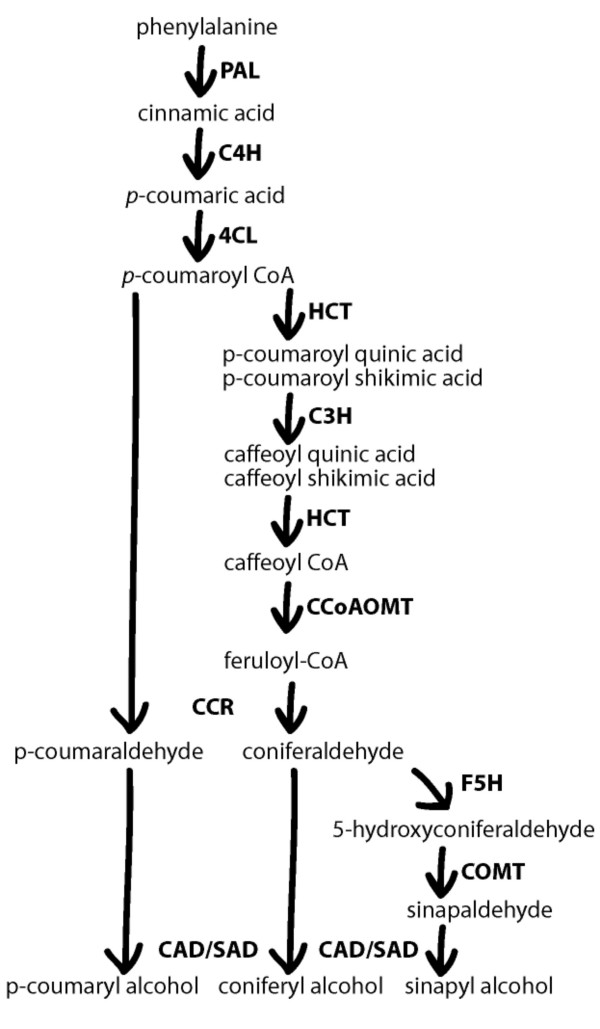
The phenyhlpropanoid pathway catalyzing the biosynthesis of monolignols in grasses (modified from Boerjan et al. 2003). Enzymes are shown in bold.

Biosynthesis of monolignols, and a variety of other secondary metabolites, is controlled by the phenylpropanoid pathway (Figure 1). The first step in the phenylpropanoid pathway is the deamination of L-phenylalanine by phenylalanine ammonia lyase (PAL) to cinnamic acid. Subsequent enzymatic steps involving the actions of cinnamate 4-hydroxylase (C4H), 4-coumarate:CoA ligase (4CL), hydroxycinnamoyl-CoA transferase (HCT), *p*-coumarate 3-hydroxylase (C3H), caffeoyl-CoA *O*-methyltransferase (CCoAOMT), cinnamoyl-CoA reductase (CCR), ferulate 5-hydroxylase (F5H), caffeic acid *O*-methyltransferase (COMT), and cinnamyl alcohol dehydrogenase (CAD) catalyze the biosynthesis of monolignols (Figure 1). In maize, one or more genes encoding each of these enzymes have been cloned [5-12]. A recent comprehensive study has shown that almost all enzymes involved in the phenylpropanoid pathway of maize, with the exception of C3H and COMT, are encoded by multigene families [8].

The four *brown-midrib *(*bm*) mutants of maize are characterized by a decreased lignin content, an altered cell wall composition, and a brown-reddish colour of leaf midribs. *bm1 *is caused by a severe decrease in CAD enzyme activity, possibly resulting from a decrease in *CAD *transcription [9,13], *bm3 *is caused by a knock-out mutation in the *COMT *gene [14,15], while the genes underlying the *bm2 *and *bm4 *mutations are unknown. Of the four known *bm *mutants, *bm3 *exhibits the strongest effect on plant phenotype, including a reduction in total lignin and an altered lignin composition [16]. A positive effect of the *bm3 *mutant has been observed on intake and digestibility of forage maize [3]. However, inferior agronomic performance such as lodging and lower biomass yield result from this mutation as well, restricting the use of *bm3 *mutants in maize breeding programs [17]. The *bm1 *mutant is also characterized by a reduction in total lignin and an altered lignin composition [16]. Characterization of genetic diversity associated with forage quality traits in genes of the phenylpropanoid pathway might facilitate identification of alleles more applicable to breeding programs.

Levels of nucleotide diversity and linkage disequilibrium (LD), and associations to forage quality traits have been reported for several genes involved in the phenylpropanoid pathway [18-21]. Due to population bottlenecks and selection, LD is generally higher among elite breeding lines than within distantly related germplasm [22]. In agreement with this, extended LD, spanning from hundreds of kb to tens of cM, has been reported among elite inbred lines [23-26]. Contrasting levels of LD have been observed between genes in the phenylpropanoid pathway. While LD decreased rapidly within few hundred bp at the *COMT *and *CCoAOMT2 *loci [20,21], LD persisted over thousands of bp at a *PAL *locus [18]. The extent of LD is relevant in the context of association (LD) mapping as it determines both the marker saturation necessary for association mapping as well as the possibility to discriminate between phenotypic effects of individual polymorphisms. The first candidate gene-based association mapping study in plants, associating individual *dwarf8 *polymorphisms with flowering time of maize [27], has been followed by numerous studies in maize [28] and other crop plants [29]. Associations between maize forage quality traits and individual polymorphisms have been reported for the *PAL*, *CCoAOMT2*, and *COMT *genes [18,20,30] as well as for the *ZmPox3 *maize peroxidase gene, putatively involved in the oxidative polymerization of monolignols [31,32]. Consequently, target sites within phenylpropanoid pathway genes for functional marker development [33] for forage quality traits have been identified.

In the present study, partial genomic sequences of *C4H*, *4CL1*, *4CL2*, *C3H*, *F5H*, and *CAD *were obtained in a set of 40 European forage maize inbred lines. Since European elite material was included in this study, LD was expected to span whole genes. Therefore, sequencing efforts were directed towards obtaining partial sequences of several genes as compared to obtaining the full sequence(s) of one/few genes, the rationale being that this would increase the number of unlinked polymorphisms available for testing by subsequent association analysis in a broader range of materials. The objectives were to (1) examine nucleotide diversity within genes, (2) examine LD within and between genes, and (3) to test for associations between individual polymorphisms and three forage quality traits.

## Results

### Phenotypic data

Analysis of variance and phenotypic correlations were published previously [18]. Mean phenotypic values for individual lines across five environments ranged from 50.33 to 63.03 for neutral detergent fiber (NDF), 67.23 to 77.98 for *in vitro *digestibility of organic matter (IVDOM), and 49.59 to 60.99 for digestibility of neutral detergent fiber (DNDF) (Table 1). The least significant differences between lines were 3.71, 2.69, and 2.70 for NDF, IVDOM, and DNDF, respectively. Heritabilities were 86.5%, 89.5%, and 92.2% for NDF, IVDOM, and DNDF, respectively.

**Table 1:** Phenotypic means for three quality-related traits across five environments. A "+" denotes that a DNA fragment of a given candidate gene has been obtained from a given line.

**Line**	**Alias**	**NDF**	**IVDOM**	**DNDF**	**C4H**	**4CL1**	**4CL2**	**CAD**	**C3H**	**F5H**
**F_AS01**	F7	58.00	74.47	60.34	+		+	+	+	
**F_AS02**	F2	52.74	74.62	56.17	+	+	+	+	+	
**F_AS03**	Ep1	50.33	76.85	59.26	+	+	+	+	+	
**F_AS04**		50.79	77.28	59.57	+	+	+	+	+	
**F_AS05**		54.17	74.89	57.43	+		+	+	+	
**F_AS06**		53.21	76.03	57.52	+	+	+	+	+	
**F_AS07**		50.38	77.98	59.48	+		+	+	+	
**D_AS08**		60.99	69.05	53.08	+	+	+	+	+	
**D_AS09**		54.60	72.26	53.00	+	+	+	+	+	+
**D_AS10**		51.89	74.27	54.59	+	+	+	+	+	+
**D_AS11**		57.08	70.10	52.21	+	+	+	+	+	+
**F_AS12**		57.91	74.32	58.94	+	+	+		+	+
**F_AS13**		57.29	74.99	59.01	+	+	+	+	+	+
**F_AS14**		56.16	72.05	54.41	+		+	+	+	+
**F_AS15**		55.47	73.12	55.81	+	+	+	+	+	+
**F_AS16**		53.02	76.21	59.56	+	+		+	+	+
**F_AS17**		61.23	69.78	54.10	+	+	+	+		+
**F_AS18**		61.43	67.95	52.18	+	+	+	+		+
**F_AS19**		59.68	72.45	57.86	+		+	+		+
**F_AS20**		52.38	72.83	54.17	+		+	+		+
**F_AS21**		51.28	76.37	57.83	+	+	+	+		+
**F_AS22**		52.39	73.02	53.03	+		+	+		+
**F_AS23**		57.26	74.2	58.34	+	+	+	+		+
**F_AS24**		51.50	77.92	60.99	+	+	+	+		+
**D_AS25**		52.92	76.65	59.41	+		+	+	+	
**D_AS26**		52.17	76.88	58.85	+	+	+	+	+	
**D_AS27**		56.79	75.11	60.43	+	+		+	+	
**D_AS28**		61.01	67.23	49.59	+	+	+	+	+	
**D_AS29**		57.96	74.49	60.16	+	+	+	+	+	
**D_AS30**		60.89	69.08	52.27	+	+	+	+	+	
**D_AS31**		63.03	71.00	58.40	+	+	+	+	+	
**D_AS32**		57.99	68.51	50.32	+	+	+	+	+	
**D_AS33**		56.14	71.56	53.02	+			+		
**D_AS34**		61.45	68.56	50.69	+	+		+		
**D_AS35**		56.68	76.06	60.95	+	+	+	+		
**D_AS36**		56.24	69.38	50.20	+	+	+	+		
**D_AS37**		59.73	72.64	58.03	+			+		
**F_AS38**		58.26	73.75	58.34	+			+		
**D_AS39**	F288	58.50	74.22	59.98	+		+			
**F_AS40**	F4	59.02	73.92	59.10	+		+	+		
**Phenotypic means**										
**Overall**		56.25	73.30	56.47						
**Flint**		55.18	74.32	57.43						
**Dent**		57.56	72.06	55.29						

Flint- and Dent lines are denoted by F_ and D_ prefixes, respectively.

NDF: neutral detergent fiber; IVDOM: *in vitro *digestibility of organic matter; DNDF: digestibility of neutral detergent fiber; *C4H*: cinnamate 4-hydroxylase; *4CL*: 4-coumarate:CoA ligase; *CAD*: cinnamyl alcohol dehydrogenase;*C3H*: *p*-coumarate 3-hydroxylase; *F5H*: ferulate 5-hydroxylase.

### Nucleotide- and haplotype diversity and selection

Partial genomic fragments were amplified for six candidate genes (names in parenthesis refer to identical genes in the MAIZEWALL database [8]): *C4H *(*C4H1*), *4CL1 *(*4CL*), *4CL2 *(not identified), *C3H *(*C3H*), *F5H *(*F5H1*), and *CAD *(Y13733). The resulting alignments were from 461 bp (*C4H*) to 1,306 bp (*4CL1*) in length and were based on 16 (*F5H*) to 40 (*C4H*) lines (Table 2). The exon-intron structure at individual loci was estimated by alignments to the mRNA sequences from which primers were developed. GENSCAN estimations supported the structures predicted by the alignments and all amplified sequences were predicted to include both coding and non-coding regions. A total of 54 SNPs were identified out of which 25 were non-redundant for discrimination of haplotypes. Total nucleotide diversity (*π*) ranged from 0.00049 at the *CAD *locus to 0.01025 at the *4CL2 *locus, and Tajima's D did not indicate selection at any of the six loci (Table 2). The number of haplotypes defined by SNPs ranged from two at the *CAD *locus (where only one SNP was identified), four at the *C4H *and *4CL1 *loci, five at the *F5H *locus, to seven at the *4CL2 *and *C3H *loci (Tables 3, 4, 5, 6, 7, 8).

**Table 2:** Summary of alignment lengths, number of genotypes per alignment, locus structure, number of haplotypes, nucleotide diversity, and Tajima's test for selection for six phenylpropanoid pathway genes in maize.

Gene	Sites (bp)	Genotypes	Locus structure/SNPs	Haplotypes	π coding	π non-coding	π total	Tajima's D
*C4H*	461	40	5' UTR: 1–33/1 1^st ^exon: 34–461/4	4	0.00355	0.00431	0.00360	1.05^NS^
*4CL1*	1,306	27	5' UTR: 1–24/1 1^st ^exon: 25–1044/17 1^st ^intron: 1045–1159/2 2^nd ^exon: 1160–1306/3	4	0.00619	0.00577	0.00615	1.17^NS^
*4CL2*	469	34	5' UTR: 1–40/3 1^st ^exon: 41–469/9	7	0.00931	0.01992	0.01025	1.86^NS^
*C3H*	607	24	Terminal exon: 1–578/7 3' UTR: 579–607/0	7	0.00251	0	0.00239	-0.72^NS^
*F5H*	1,220	16	Exon: 1–76/5 Intron: 77–1220/2	5	0.02905	0.00076	0.00383	0.72^NS^
*CAD*	564	38	Terminal exon: 1–378/1 3' UTR: 379–564/0	2	0.00072	0	0.00049	0.21^NS^

*C4H*: cinnamate 4-hydroxylase; *4CL*: 4-coumarate:CoA ligase; *C3H*: *p*-coumarate 3-hydroxylase; *F5H*: ferulate 5-hydroxylase; *CAD*: cinnamyl alcohol dehydrogenase

^NS ^Not significant.

**Table 3:** Haplotypes based on single nucleotide polymorphisms (SNPs) in the *cinnamate 4-hydroxylase (C4H) *gene of maize and average phenotypic values of lines included in individual haplotypes. Numbers denote bp position of individual SNPs in the alignment.

	31	50	112	140	161	Lines (Total = 40)	NDF	IVDOM	DNDF
H_1	G	A	C	G	G	AS01, 02, 07–11, 23–37, 40	56.8	72.9	56.1
H_2	A	G	G	.	T	AS17, 20, 39	57.4	72.3	56.1
H_3	.	.	G	.	.	AS03, 12, 13	55.2	75.4	59.1
H_4	.	G	G	C	.	AS04-06, 14–16, 18, 19, 21, 22, 38	55.1	73.9	56.7

NDF: neutral detergent fiber; IVDOM: *in vitro *digestibility of organic matter; DNDF: digestibility of neutral detergent fiber

**Table 4:** Haplotypes based on single nucleotide polymorphisms (SNPs) in the *4-coumarate:CoA ligase 1 (4CL1) *gene of maize and average phenotypic values of lines included in individual haplotypes. Numbers denote bp position of individual SNPs in the alignment.

	10	31	35	37	56	182	191	218	431	457	481	502	523	675	718	817	844	911	1119	1154	1236	1260	1284	Lines (Total = 27)	NDF	IVDOM	DNDF
	s	s	s	s													s	s		s		s	s				
H_1	C	C	T	C	G	G	G	C	T	T	C	T	C	G	T	C	T	G	G	A	G	C	A	AS02, 04, 15, 17, 18, 21, 23, 24, 36	55.3	73.4	56.1
H_2	.	.	.	.	A	A	C	A	G	C	G	C	T	A	C	G	.	.	C	.	A	.	.	AS03, 06, 08–13, 16, 26–30, 34, 35	56.5	73.2	56.3
H_3	.	.	.	.	.	A	C	A	G	C	G	C	T	A	C	G	.	.	C	.	A	.	.	AS32	58.0	68.5	50.3
H_4	T	T	G	T	.	.	.	.	G	C	G	C	T	.	C	G	C	T	C	G	.	T	C	AS31	63.0	71.0	58.4

NDF: neutral detergent fiber; IVDOM: *in vitro *digestibility of organic matter; DNDF: digestibility of neutral detergent fiber

s: singleton

**Table 5:** Haplotypes based on single nucleotide polymorphisms (SNPs) in the *4-coumarate:CoA ligase 2(4CL2) *gene of maize and average phenotypic values of lines included in individual haplotypes. Numbers denote bp position of individual SNPs in the alignment.

	11	22	32	72	112	123	132	162	192	217	372	460	Lines (Total = 34)	NDF	IVDOM	DNDF
			s													
H_1	A	A	G	G	A	G	T	G	C	A	A	C	AS01, 04, 06, 14, 15, 18, 22, 24	54.9	74.0	56.7
H_2	G	.	.	.	G	.	C	.	T	G	C	.	AS03, 08–11, 17, 19–21, 30, 31, 35, 36	56.6	72.3	55.2
H_3	G	T	.	A	G	T	C	A	.	.	.	.	AS12, 13	57.6	74.7	59.0
H_4	.	.	.	.	.	.	.	.	.	.	.	A	AS02	52.7	74.6	56.2
H_5	G	.	.	.	.	.	.	.	.	.	C	A	AS07, 23, 25, 26	53.2	76.4	59.0
H_6	G	T	.	A	G	T	C	A	.	.	C	A	AS05, 28, 29, 32, 39	57.9	71.9	55.5
H_7	G	.	A	.	G	.	C	.	.	.	.	.	AS40	59.0	73.9	59.1

NDF: neutral detergent fiber; IVDOM: *in vitro *digestibility of organic matter; DNDF: digestibility of neutral detergent fiber

s: singleton

**Table 6:** Haplotypes based on single nucleotide polymorphisms (SNPs) in the *p-coumarate 3-hydroxylase (C3H) *gene of maize and average phenotypic values of lines included in individual haplotypes. Numbers denote bp position of individual SNPs in the alignment.

	8	59	294	452	479	548	Lines (Total = 24)	NDF	IVDOM	DNDF
H_1	A	G	G	T	G	G	AS01, 08–13, 25–27, 29–32	57.1	72.9	56.5
H_2	.	.	C	C	C	.	AS14	56.2	72.1	54.4
H_3	G	T	.	.	.	.	AS15, 16	54.2	74.7	57.7
H_4	.	.	.	C	T	.	AS02, 05, 07	52.4	75.8	57.7
H_5	.	.	C	.	C	A	AS28	61.0	67.2	49.6
H_6	.	.	.	.	T	.	AS03, 06	51.8	76.4	58.4
H_7	.	.	.	.	C	A	AS04	50.8	77.3	59.6

NDF: neutral detergent fiber; IVDOM: *in vitro *digestibility of organic matter; DNDF: digestibility of neutral detergent fiber

**Table 7:** Haplotypes based on single nucleotide polymorphisms (SNPs) in the *ferulate 5-hydroxylase (F5H) *gene of maize and average phenotypic values of lines included in individual haplotypes. Numbers denote bp position of individual SNPs in the alignment.

	5	6	11	56	65	97	610	Lines (Total = 16)	NDF	IVDOM	DNDF
							s				
H_1	C	G	G	T	T	C	G	AS12-15, 17	57.6	72.9	56.5
H_2	.	.	.	.	C	.	.	AS16, 18, 19	58.0	72.2	56.5
H_3	G	C	.	.	.	.	.	AS20-23	53.3	74.1	55.8
H_4	G	C	.	.	C	.	C	AS24	51.5	77.9	61.0
H_5	.	.	C	C	C	T	.	AS09-11	54.5	72.2	53.3

NDF: neutral detergent fiber; IVDOM: *in vitro *digestibility of organic matter; DNDF: digestibility of neutral detergent fiber

s: singleton

**Table 8:** Haplotypes based on single nucleotide polymorphisms (SNPs) in the *cinnamyl alcohol dehydrogenase (CAD) *gene of maize and average phenotypic values of lines included in individual haplotypes. Numbers denote bp position of individual SNPs in the alignment.

	366	Lines (Total = 38)	NDF	IVDOM	DNDF
H_1	C	AS01, 02, 04–11, 13–16, 22–38, 40	56.2	73.4	56.4
H_2	A	AS03, 17–21	56.1	72.7	55.9

NDF: neutral detergent fiber; IVDOM: *in vitro *digestibility of organic matter; DNDF: digestibility of neutral detergent fiber

### Intra- and inter-locus linkage disequilibrium

**Figure 2:**
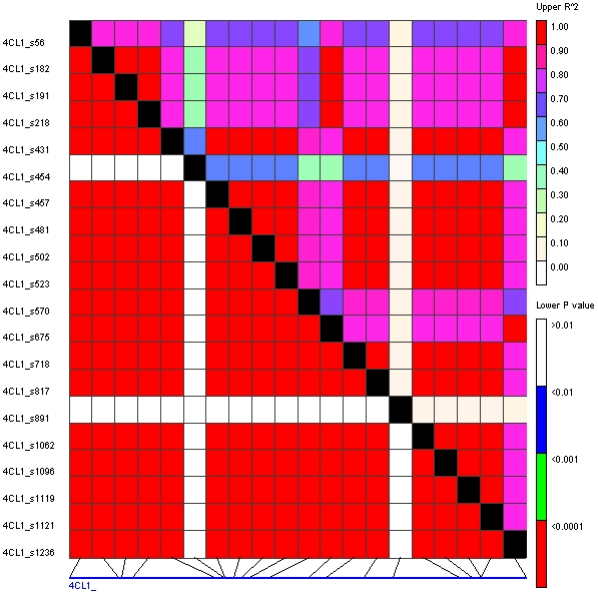
Linkage disequilibrium at the 4CL1 locus. Numbers on the left column denote bp position in the alignment. Indel polymorphisms are identified at positions 454, 570, 891, 1062, and 1121 while the remaining polymorphisms are SNPs.

**Figure 3:**
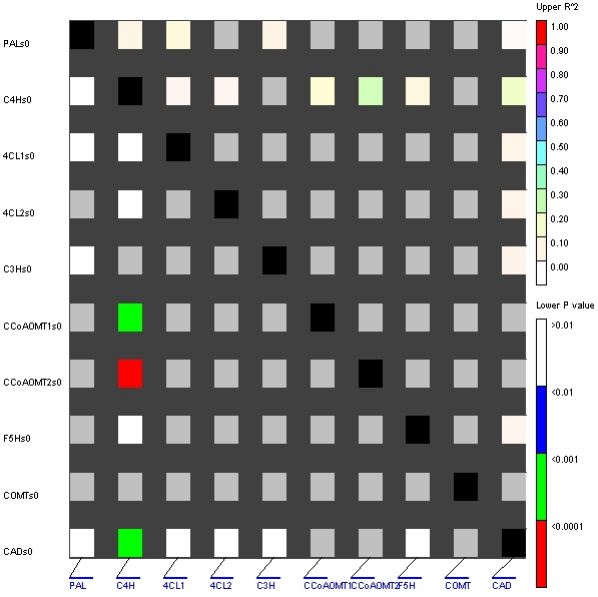
Linkage disequilibrium between haplotypes of individual genes in the phenylpropanoid pathway in maize.

Extended LD was identified at the *4CL1 *locus at which all polymorphisms, with the exception of two 1-bp deletions, were in high LD (P > 0.001) across the entire amplified sequence (~1.3 kb; Figure 2). At the *C4H*, *C3H*, *4CL2*, and *F5H *loci, breakdown of LD was observed within ~200 bp. Inter-locus LD was examined by estimating LD between SNP haplotypes of the six loci as well as *PAL *(32 lines), *COMT *(42 lines), *CCoAOMT1 *(40 lines) and *CCoAOMT2 *(34 lines) ([18,21], unpublished results). This revealed that *C4H *were in high (P < 0.0001) LD with *CCoAOMT2 *and intermediate (P < 0.001) LD with *CCoAOMT1 *and *CAD*. Significant LD was not observed between any other pairs of loci (Figure 3). Examining LD between individual SNPs at these three loci pinpointed that a single non-synonymous SNP, changing the 27^th ^amino acid of the C4H enzyme from Threonine to Serine, was in high LD with several SNPs at the *CCoAOMT1 *and *CCoAOMT2 *locus, respectively (data not shown).

### Population structure and marker-trait associations

Within *Structure *we evaluated whether the 40 lines constitute one, two, three, or four subpopulations, respectively. Two subpopulations (*K *= 2) was the most likely scenario (results not shown). Most lines were estimated to be > 99% Flint or Dent, in agreement with pedigree information. Under the assumption of two subpopulations, four lines showed approximate 3:1(AS27 and AS29) or 1:3 (AS34 and AS39) ratios of genetic background of Dent:Flint.

The estimated population structure matrix was included in the association analysis, performed as GLM analysis in *TASSEL*. At the *4CL1 *locus a 1-bp indel was associated with NDF and IVDOM (Table 9). The insertion allele was present in only one line (AS18), which exhibits NDF = 61.43 compared to an overall mean of 56.25, and IVDOM = 67.95 compared to an overall mean of 73.30 (Table 1). At the *C3H *locus, a non-synonymous G/C SNP at position 294 of the alignment was associated with both IVDOM and DNDF. The C allele was present in two lines (AS14 and AS28). While IVDOM and DNDF values for AS14 are slightly below the overall means, AS28 exhibits the lowest overall values for both IVDOM and DNDF, 67.23 and 49.59, respectively (Table 1). At the *F5H *locus, two non-synonymous SNPs, at positions 5 and 6 and in complete LD, were associated with NDF. The G and C allele, respectively, of these two C/G SNPs were present in lines AS20 to AS24. The mean NDF value of these five lines is 52.96 compared to an overall mean of 56.25. The line AS23 is differing from the other four lines in this haplotype as it exhibits an NDF value above the overall mean (Table 1). In addition, two SNPs in the intron region of *F5H *were associated with DNDF (C/G SNP, position 610) and NDF (C/T SNP, position 817). At position 610 a singleton SNP was present in line AS24, exhibiting the highest overall DNDF value (Table 1). At position 817, the C allele was present in lines AS14, AS15, and AS20 to AS22, the mean of these lines being below the overall mean of NDF. It should be noted that for *F5H*, only 16 lines was included in the sample. In addition, the SNP at position 817 was genotyped for only 13 lines due to an indel polymorphism in this region. Consequently, this SNP was not included in the haplotype overview (Table 7). No associations with forage quality traits were detected for the *4CL2*, *C4H*, and *CAD *gene fragments.

**Table 9:** Associations between individual polymorphisms (denoted by position in the alignment) and forage quality traits. The analyses were performed including overall population structure (GLM) and both overall population structure and relative kinship (MLM).

Gene	Position	Polymorphism	Assoc. trait	Identified by
4CL1	810	Frameshift indel^1^	NDF	GLM*
			IVDOM	GLM**, MLM*
C3H	294	Gly to Arg SNP	IVDOM	GLM*, MLM*
			DNDF	GLM*
F5H	5–6	Pro to Arg SNPs^2^	NDF	GLM*
	610	Intron SNP^3^	DNDF	GLM*
	817	Intron SNP	NDF	GLM*, MLM*

*4CL*: 4-coumarate:CoA ligase; *C3H*: *p*-coumarate 3-hydroxylase; *F5H*: ferulate 5-hydroxylase; NDF: neutral detergent fiber; IVDOM: *in vitro *digestibility of organic matter; DNDF: digestibility of neutral detergent fiber

*P < 0.05

** P < 0.01

^1^Singleton indel, a one-bp deletion identified in only one line (AS18)

^2^SNPs at position 5 and 6 are in complete LD

^3^Singleton SNP, one allele present in only one line (AS24)

The associations identified by GLM were validated by the MLM method, which in addition to overall population structure also corrects for finer scale relative kinship. By MLM, significant associations (P < 0.05) of the *4CL1 *indel with IVDOM, the *C3H *SNP with IVDOM, and one *F5H *intron SNP with NDF were identified (Table 9). No association to DNDF was detected when correcting for both overall population structure and relative kinship. Controlling for multiple testing by the FDR method requires P < 0.005 to reject the hypothesis of no association. One association, identified by GLM analysis, satisfied this constraint: the association of the 1 bp frameshift indel in *4CL1 *with IVDOM (P = 0.0017).

## Discussion and conclusion

### Nucleotide diversity and linkage disequilibrium in the phenylpropanoid pathway

In the present study, the partial genomic sequence of six genes putatively involved in the phenylpropanoid pathway has been obtained for 16 to 40 inbred lines of European maize. Population bottlenecks and selection are expected to decrease nucleotide diversity and increase LD at a given locus [22,23]. While selection was not indicated at any of the six loci (Table 2) nucleotide diversity (*π*) varied considerably between loci, ranging from 0.00049 at the *CAD *locus to 0.01025 at the *4CL2 *locus. Comparable levels of nucleotide diversity have been reported for other genes of the phenylpropanoid pathway within a similar and overlapping set of lines [18,21] as well as within a more diverse set of lines [20]. Also, a comprehensive study of six genes of the starch pathway of maize revealed similar levels of diversity [34]. Nucleotide diversity at the *CAD *locus is exceptionally low as compared to other phenylpropanoid pathway genes, with only one SNP identified across 38 genotypes (Table 2). While the *CAD *sequence is relatively short (~0.5 kb), several SNPs were identified within fragments of similar length for other genes (Table 2).

Levels of LD varied between loci, spanning the full *4CL1 *sequence (~1.3 kb) while decaying within few hundred bps at the *C4H*, *C3H*, *4CL2*, and *F5H *loci. Due to population bottlenecks and selection, LD can be expected to be higher among elite breeding lines as compared to more distantly related germplasm. In agreement with this, a rapid LD decay (*r*^2 ^< 0.1 within few hundred bps) has been reported for several loci in diverse sets of maize germplasm [35,36] while extended LD, up to tens of cM, has been reported among elite inbred lines [23-26]. However, extended LD was also observed at the *sugary1 *locus in a set of diverse germplam [35] indicating considerable variation in LD between loci regardless of sampled plant material. Varying levels of LD have previously been observed between genes of the phenylpropanoid pathway, decaying within few hundred bps for *CCoAOMT2 *and *COMT *[20,21] while spanning more than 3.5 kb at the *PAL *locus [18], supporting that LD decay is differentiating more between loci than between samples of different origin, e.g., between elite breeding lines and more distantly related germplams. In agreement with this, LD decay at the *COMT *locus was similar between a diverse set of lines (*r*^2 ^= 0.2 within ~250 bp) [20] and a set of elite European breeding lines (*r*^2 ^= 0.2 within ~500 bp) [21].

Varying levels of nucleotide diversity and LD between loci could reflect different levels of constraints put on individual loci by selection. It might also be speculated that these parameters would be influenced by length of exons and introns contained in individual gene amplicons. However, levels of nucleotide diversity and LD did not seem to be correlated to exon:intron proportions of individual genes (Table 2 and data not shown). Nucleotide diversity at the *CAD *locus, encoding the enzyme catalyzing the last step in monolignol biosynthesis, the reduction of *p*-hydroxycinnamaldehydes into their respective alcohols, is found to be exceptionally low, with only one SNP identified across the ~0.5 kb examined in this study. It should be noted that the level of nucleotide diversity identified here might not be indicative for the *CAD *locus as a whole. A recent comprehensive study of gene expression in relation to cell wall biosynthesis in maize identified a total of seven *CAD *gene family members, of which the one examined here was highly expressed in internodes [8]. In addition, reduced CAD enzyme activity and altered lignin content and structure were observed in the *bm1 *mutant [9], most likely resulting from decreased expression of this and/or other *CAD *genes [9,13]. Thus, an important role in lignification is indicated for this *CAD *gene, suggesting selection against detrimental mutations at this locus.

While nucleotide diversity at the *4CL1 *locus was found to be ~10 fold higher than for the *CAD *gene, all SNPs across the *4CL1 *locus (~1.3 kb) were in high LD (Figure 2). This is comparable to the situation at the *PAL *locus, at which all informative polymorphisms were in complete LD across ~2.5 kb within an overlapping sample of lines [18]. PAL is the first enzyme in several phenylpropanoid pathways, catalyzing the production of a number of phenylpropanoids, including monolignols, from phenylalanine. In *Arabidopsis *it has been observed that *PAL *mutants were affected not only in the monolignol pathway, but that also carbohydrate- and amino acid metabolisms were altered [37]. While the 4CL enzyme is further downstream in the phenylpropanoid pathway, it is before the branching of the pathway into monolignol-, flavonoid- and other biosynthetic pathways. The *4CL1 *gene investigated in the present study is highly expressed in leaves and young stems of maize [8], indicating an important function of the enzyme in these tissues. Thus, functional constraints of the enzyme might restrict recombination rates at the gene, resulting in the extended LD observed at the *4CL1 *locus.

While LD decay was rapid within the *C4H *gene, a single non-synonymous SNP in *C4H *was in high LD with several SNPs in the *CCoAOMT1 *and *CCoAOMT2 *genes, respectively. While *C4H *is located on chromosome 8 (unpublished results), the *CCoAOMT1 *and *CCoAOMT2 *genes are located on chromosomes 6 and 9, respectively [20]. It could be speculated that *C4H*, *CCoAOMT1*, and *CCoAOMT2 *are epistatically interacting, i.e., particular allelic variants leading to altered C4H enzymes are dependent on specific allelic properties at the two *CCoAOMT *loci. An expression-QTL for cell wall biosynthesis genes has been identified at bin 9.04 [38] near *CCoAOMT2 *at bin 9.02 [20]. Thus, given the limited precision of QTL mapping experiments, it could be speculated that *CCoAOMT2 *is involved, directly or indirectly, in the regulation of several cell wall biosynthesis genes.

### Association of genetic variation in the phenylpropanoid pathway and forage quality

Previous studies have identified associations between phenylpropanoid pathway genes and forage quality traits [18,20,30]. In the present study we have identified associations between polymorphisms in *4CL1*, *C3H*, and *F5H *and NDF, IVDOM, and/or DNDF, of which DNDF is the most relevant trait in relation to forage quality. No associations were detected for *4CL2*, *C4H*, and *CAD *polymorphisms. When correcting for multiple testing the association between *4CL1 *and IVDOM remained significant. The *4CL1 *gene investigated in the present study is homologous to the *4CL1 *of *Arabidopsis *[39] and is highly expressed in leaves and young stems of maize [8]. In *Arabidopsis*, *4CL1 *has been shown to be involved in the biosynthesis of lignin, antisense lines being depleted in G monolignol units [40]. In the present study, a 1-bp indel in *4CL1 *was found to be associated with IVDOM by both GLM and MLM. The insertion allele of this indel is present in only one line, AS18, which exhibits the second lowest overall value of IVDOM. The insertion results in a frameshift in the first exon, introducing a premature stop codon four amino acids downstream the insertion. It is thus likely that this indel directly influences the function of the 4CL1 enzyme. In relation to association analysis the situation of a single phenotypically extreme individual is a potential problem. This individual might show numerous specific mutations, which consequently would show association to the phenotype if tested. In the present study, associations based on one/few phenotypically extreme individuals could explain why associations are identified to IVDOM and not to DNDF, and *vice versa*, in spite of these two traits being highly correlated [18]. Including population structure and kinship into association analysis reduces the number of false positive associations. However, associations based on single phenotypically extreme individuals should be considered with caution and validated in broader plant material.

While not significant when controlling for multiple testing, a non-synonymous SNP in the terminal exon of *C3H *was associated with IVDOM by both GLM and MLM. The C allele of this G/C SNP was identified in two lines of which AS28 exhibits the lowest overall values of both IVDOM and DNDF. In *Arabidopsis *[41] and maize [8], a single *C3H *gene has been identified, which in maize is expressed in relatively low levels in different tissues [8]. A reduced transcription of *C3H *has been shown to affect lignin content and composition in both *Arabidopsis *[42] and alfalfa [43]. Moreover, alfalfa lines down-regulated in *C3H *transcription exhibited increased *in vitro *dry matter digestibility (IVDMD) [44]. Given a similar function of the C3H enzyme in maize, allelic variation at this locus might directly affect lignin content and composition and in turn digestibility of the maize cell wall.

At the *F5H *gene, a SNP in the intron was associated with NDF by both GLM and MLM, but not when controlling for multiple testing. Two *F5H *genes have been identified in both *Arabidopsis *[45,46] and maize [8,11]. In *Arabidopsis *[47] and alfalfa [44], plants deficient in *F5H *transcription exhibit an altered composition of lignin. However, an effect on NDF or IVDMD was not observed in *F5H *down-regulated lines of alfalfa [44]. In maize, the *F5H *gene analyzed in this study is highly expressed in young stems and in leaves [8]. It can be questioned if a SNP in the intron region has a causative effect on phenotypic traits. However, this SNP might be in LD with causative variation in other regions of the ORF.

One of the factors greatly affecting the outcome of the association analysis is the choice of method for testing associations (Table 9) [28,48]. By correcting for overall population structure (Q) ten significant associations were identified, five of which were confirmed when including correction for relative kinship (K). No additional associations were identified when correcting for both Q and K as compared to when correcting only for Q. Not surprisingly, a greater number of associations were identified when no correction for population structure was made (data not shown). It has been shown that the number of false positive associations is reduced when controlling Q and, to a greater extend, when controlling for both Q and K [48]. However, both trait values and causative polymorphisms might be confounded with population structure, e.g., between Flint and Dent lines. In consequence, true positive associations might not be identified by association analysis when considering population structure. To avoid this, it might be beneficial (if possible) to ensure an even distribution of trait values within and between subpopulations in the plant genotype sample employed for association analysis. Independent of the method, sample size is an important factor and, in the present study, a limiting factor in relation to association analyses. Consequently, the associations reported here should be considered indicative and validated in a larger sample of lines before applied in, e.g., breeding programs. In addition, due to the limited extent of LD at four out of six genes, full-length sequences of these genes would likely increase the number of unlinked polymorphisms to be tested for associations.

### Deriving functional markers for forage quality traits

With the results presented here, genes encoding most of the enzymes in the phenylpropanoid pathway in maize have been tested for association with forage quality traits. The *PAL *gene was investigated in a set of 32 European elite inbred lines, overlapping with the lines used in this study [18]. A 1-bp deletion in the second exon of *PAL*, introducing a premature stop codon, was associated with high IVDOM. Two *CCoAOMT *genes have been investigated in a set of 34 diverse lines used in both European and US breeding programs [20]. While no associations were detected for *CCoAOMT1*, an SSR-like insertion in the first exon of *CCoAOMT2 *was associated with an increase in cell wall digestibility. For the *COMT *gene, indel polymorphisms in the intron region have been associated with cell wall digestibility in two different sets of lines, one of which are overlapping with the set employed in the present study [20,30]. Specifically, a 1-bp deletion in a putative splice site recognition site was associated with high cell wall digestibility [20]. Likewise, a MITE insertion in the second exon of the *ZmPox3 *gene, encoding a peroxidase putatively involved in monolignol polymerization, was associated with high cell wall digestibility [32].

Combining previous results with the results reported in the present study, it seems likely that several genes of the phenylpropanoid pathway can be considered candidate genes for deriving functional markers for forage quality. In addition, it is indicated that useful variation in these genes can be identified even within elite breeding lines of maize, although alleles with larger effects on phenotype might be mined from a broader and larger sample of lines. Polymorphisms in genes encoding enzymes downstream in the phenylpropanoid pathway have been indicated to increase cell wall digestibility [20,30,32], while similar polymorphisms have been associated with increase and decrease in IVDOM for *PAL *[18] and *4CL1 *(Table 9), respectively. It could be speculated that genes more downstream in the phenylpropanoid pathway (Figure 1) would make more suitable targets for functional marker development in relation to digestibility of the cell wall. Such genes might be more specific to lignin biosynthesis as compared to genes acting earlier in the phenylpropanoid pathway, which could possibly affect several pathways as illustrated by *PAL *in *Arabidopsis *[37]. However, the recent identification of gene families in most genes of the phenylpropanoid pathway in maize [8] might suggest that specialization towards biosynthesis of lignin occur earlier in the phenylpropanoid pathway than previously assumed.

## Methods

### Plant materials and phenotypic analyses

A collection of 40 maize inbred lines consisting of 22 Flint and 18 Dent lines were included in this analysis. The line collection is identical to the one published previously [18], with the addition of eight lines. Thirty-five lines were from the current breeding program of KWS Saat AG and five lines were from the public domain (AS01, AS02, AS03, AS39, and AS40 identical to F7, F2, EP1, F288, and F4, respectively; Table 1). This collection of lines was selected based on DNDF values to represent a broad range of variability for this trait in central European germplasm employed in forage maize breeding. The included lines were derived from different Flint and Dent breeding populations, respectively, and are not related by descent apart from lines AS20 and AS21 which form an isogenic line pair differing for DNDF. The inbred lines were evaluated in Grucking (sandy loam) in 2002, 2003, and 2004, and in Bernburg (sandy loam) in 2003 and 2004. The experiments included 49 entries in a 7 × 7 lattice design with two replications. Plots consisted of single rows, 0.75 m apart and 3 m long with a total of 20 plants. About 50 days after flowering the ears were manually removed and the stover was chopped. Approximately 1 kg of the material was collected and dried at 40°C. The stover was ground to pass through a 1 mm sieve. Quality analyses were performed with near infrared reflectance spectroscopy (NIRS) based on previous calibrations on the data of 300 inbred lines (unpublished results). The following data were recorded: NDF [49], IVDOM [50], and DNDF given by the formula DNDF = 100 - (100 - IVDOM)/(NDF × DM/OM/100) where DM is dry matter content and OM is organic matter content of the sample.

### DNA isolation, PCR amplification, and DNA sequencing

Plants were grown for DNA isolation in the greenhouse and leaves were harvested at three weeks after germination. Genomic DNA was extracted from the leaves using the Maxi CTAB method [51]. Polymerase chain reaction (PCR) primers were developed for six candidate genes (*C4H*, *4CL1*, *4CL2*, *C3H*, *F5H*, and *CAD*) based on maize mRNA sequences identified in GenBank (Table 10) by BLASTing [52] known phenylpropaniod pathway genes. PCR reactions contained 20 ng genomic DNA, primers (200 nM), dNTPs (200 μM), 1 M Betain and 2 units of Taq polymerase (Peqlab, Erlangen, Germany) in a total reaction volume of 50 μl. A touchdown PCR program was applied as follows: an initial denaturation step at 95°C for 2 min, 15 amplification cycles: 45 sec at 95°C; 45 sec at 68°C (minus 0.5°C per cycle), 2 min at 72°C, followed by 24 amplification cycles: 45 sec at 95°C; 45 sec at 60°C, 2 min at 72°C, and a final extension step at 72°C for 10 min. Products were separated by gel electrophoresis on 1.5% agarose gels, visualized by ethidium bromide staining and photographed using an eagle eye apparatus (Herolab, Wiesloch, Germany).

**Table 10:** The maize mRNA templates (GenBank accession numbers) from which primers were developed for amplifying genomic fragments of six phenylpropanoid pathway genes.

Gene	Template	Primers
*C4H*	AY104175	F: 5' AAA CCA CAC ACC CCA CCT AC
		R: 5' GGT CCT TCC TCA CGT CCT C
*4CL1*	AX204867	F: 5' ATC CAG GTC CAG CTC CAC CAA
		R: 5' TGC CTC CGG GTT GTT GAG GTA
*4CL2*	AX204868	F: 5' AAC GTT ACC TGC CCG ACA T
		R: 5' CTT GGC GAT CTC CAC CAC
*C3H*	AY107051	F: 5' GGA TCG TCC ACA ACG GCA TCA
		R: 5' GGG AAC GCA GCA GAT GCC AGG AC
*F5H*	AX204869	F: 5' ACA TGC TCG CCT TCT TCG C
		R: 5' GCG CAT GGC GTC AGT ACA AGG
*CAD*	AJ005702	F: 5' ACT CGC TGG ACT ACA TCA TCG ACA CG
		R: 5' GCT GGT GAG ACT GAC ACC AC

*C4H*: cinnamate 4-hydroxylase; *4CL*: 4-coumarate:CoA ligase; *C3H*: *p*-coumarate 3-hydroxylase; *F5H*: ferulate 5-hydroxylase; *CAD*: cinnamyl alcohol dehydrogenase

Amplicons were purified using QiaQuick spin columns (Qiagen, Valencia, USA) according to the manufacturers instructions, and sequenced directly using internal sequence specific primers and the Big Dye1.1 dye-terminator sequencing kit on an ABI 377 (PE Biosystems, Foster City, USA). Electropherograms of overlapping sequencing fragments were manually edited using the software package Sequence Navigator version 1.1 from PE Biosystems. Full alignments were built up using default settings of the Clustal program version 1.8 [53] followed by manual refinement to minimize the number of gaps.

### Analysis of sequence data

The exon-intron structure of the amplified genomic sequences was estimated by alignment to the mRNA sequences used for primer development (Table 10) and validated by the GENSCAN web server at the Massachusetts Institute of Technology [54].

Nucleotide diversity (*π*), the average number of nucleotide differences per site between two sequences was estimated by using DNASP Version 4.10 [55]. Indel polymorphisms were excluded from the estimates of *π *. Tajima's D statistic [56] was also estimated by DNASP to test for selection at individual loci. LD between pairs of polymorphic sites (SNPs and indels, excluding singletons) within and between loci was estimated by the TASSEL software, version 1.9.0 [27,57]. Various measurements for LD have been developed [58] of which squared allele frequency correlations (*r*^2^) [59] were chosen for our calculations. The significance of LD between sites was tested by Fisher's exact test. For the estimation of inter-locus LD, the alignments of *PAL*, *COMT*, *CCoAOMT1*, and *CCoAOMT2 *([18,21], unpublished results) were included.

### Population structure and association analysis

Lines were genotyped with 101 simple sequence repeat markers (SSRs) providing an even coverage of the maize genome. The employed SSR markers are publicly available [60]. Population structure was inferred from the SSR data by the *Structure *2.0 software [61,62]. *Structure *applies a Bayesian clustering approach to group individual lines in subpopulations based on marker profiles. A Q matrix is produced that lists the estimated membership coefficients for each individual in each subpopulation. A burn-in length of 50.000 followed by 50.000 iterations was used. The Admixture model was applied with independent allele frequencies. Data were defined as haploid.

Association analysis was carried out as a general linear model (GLM) analysis in *TASSEL *to test for associations between individual polymorphisms and mean phenotypic values across five environments (Table 1). The Q matrix produced by *Structure *was included as covariate in the analysis to control for populations structure. All polymorphisms (including singletons) were tested and the P-value for individual polymorphisms was estimated based on 10,000 permutations of the dataset. Associations were further tested by the unified mixed model method for association mapping (MLM) in *TASSEL *[48]. The MLM simultaneously accounts for overall population structure (Q) and finer scale relative kinship (K). Loiselle kinship coefficients [63] between lines (a K matrix) were estimated by the SPAGeDI software [64] based on the SSR data mentioned above. Negative values between two individuals in the K matrix were set to 0 as a negative value indicates that two individuals are less related that random individuals [64]. The diagonal of the K matrix were assigned the value 2. The False Discovery Rate (FDR) method [65] was applied to correct for multiple testing.

## List of abbreviations

4CL: 4-coumarate:CoA ligase, C3H: *p*-coumarate 3-hydroxylase, C4H: cinnamate 4-hydroxylase, CAD: cinnamyl alcohol dehydrogenase, CCoAOMT: caffeoyl-CoA *O*-methyltransferase, COMT: caffeic acid *O*-methyltransferase, DNDF: digestibility of neutral detergent fiber, F5H: ferulate 5-hydroxylase, indel: insertion-deletion polymorphism, IVDOM: *in vitro *digestibility of organic matter, LD: linkage disequilibrium, NDF: neutral detergent fiber, PAL: phenylalanine ammonia-lyase, SNP: single-nucleotide polymorphism

## Authors' contributions

JRA performed the data analysis and prepared the manuscript. IZ carried out allele sequencing. GW contributed to experimental design. BD and JE provided phenotypic data. MO provided the SSR data and together with GW contributed to experimental design. TL coordinated the project and together with JRA prepared the manuscript. All authors read and approved the final manuscript.
